# Seven Glycolysis-Related Genes Predict the Prognosis of Patients With Pancreatic Cancer

**DOI:** 10.3389/fcell.2021.647106

**Published:** 2021-04-01

**Authors:** Han Nie, Cancan Luo, Kaili Liao, Jiasheng Xu, Xue-Xin Cheng, Xiaozhong Wang

**Affiliations:** ^1^Department of Vascular Surgery, The Second Affiliated Hospital of Nanchang University, Nanchang, China; ^2^Department of Hematology, The Second Affiliated Hospital of Nanchang University, Nanchang, China; ^3^Jiangxi Province Key Laboratory of Laboratory Medicine, Department of Clinical Laboratory, The Second Affiliated Hospital of Nanchang University, Nanchang, China; ^4^Jiangxi Province Key Laboratory of Laboratory Medicine, The Second Affiliated Hospital of Nanchang University, Nanchang, China

**Keywords:** glycolysis-related genes, pancreatic ductal carcinoma, prognosis, model, predict

## Abstract

**Objectives:**

To identify the key glycolysis-related genes (GRGs) in the occurrence and development of pancreatic ductal carcinoma (PDAC), and to construct a glycolysis-related gene model for predicting the prognosis of PDAC patients.

**Methodology:**

Pancreatic ductal carcinoma (PDAC) data and that of normal individuals were downloaded from the TCGA database and Genotype-Tissue Expression database, respectively. GSEA analysis of glycolysis-related pathways was then performed on PDAC data to identify significantly enriched GRGs. The genes were combined with other patient’s clinical information and used to construct a glycolysis-related gene model using cox regression analysis. The model was further evaluated using data from the validation group. Mutations in the model genes were subsequently identified using the cBioPortal. In the same line, the expression levels of glycolysis related model genes in PDAC were analyzed and verified using immunohistochemical images. Model prediction for PDAC patients with different clinical characteristics was then done and the relationship between gene expression level, clinical stage and prognosis further discussed. Finally, a nomogram map of the predictive model was constructed to evaluate the prognosis of patients with PDAC.

**Results:**

GSEA results of the training set revealed that genes in the training set were significantly related to glycolysis pathway and iconic glycolysis pathway. There were 108 differentially expressed GRGs. Among them, 29 GRGs were closely related to prognosis based on clinical survival time. Risk regression analysis further revealed that there were seven significantly expressed glycolysis related genes. The genes were subsequently used to construct a predictive model. The model had an AUC value of more than 0.85. It was also significantly correlated with survival time. Further expression analysis revealed that CDK1, DSC2, ERO1A, MET, PYGL, and SLC35A3 were highly expressed in PDAC and CHST12 was highly expressed in normal pancreatic tissues. These results were confirmed using immunohistochemistry images of normal and diseases cells. The model could effectively evaluate the prognosis of PDAC patients with different clinical characteristics.

**Conclusion:**

The constructed glycolysis-related gene model effectively predicts the occurrence and development of PDAC. As such, it can be used as a prognostic marker to diagnose patients with PDAC.

## Introduction

Pancreatic ductal carcinoma (PDAC) is one of the most fatal malignant tumors. It is ranks fourth in cancer related deaths in the United States. Its 5-year survival rate is as low as 6% (Siegel et al., 2019). The low survival rate of PDAC is attributed to the location of the pancreas (adjacent to many organs) which makes it difficult to detect pancreas anomalies by routine examination. As such, most PDAC patients have metastases by the time they are diagnosed. Currently, surgical resection is the main treatment method for early PDAC. The 5-year survival rate of PDAC patients undergoing surgical resection is only 20%. Combination chemotherapy such as FOLFIRINOX combination therapy that consists of folic acid, fluorouracil, irinotecan and oxaliplatin (Conroy et al., 2011), and combined use of gemcitabine and nab- paclitaxel (von Hoff et al., 2013) have become the preferred treatment option for patients with advanced PDAC.

However, the survival time of patients using these combination regimens is still very low. The survival time averages at only 11 months when FOLFIRINOX is used and 8 months when there is combined use of gemcitabine and nab-paclitaxel. There is therefore a need to identify early diagnosis and more effective treatment strategies.

The metabolic processes of tumor environments have gradually become research hot spots in tumor research and treatment in recent decades (Vander Heiden et al., 2009; Jones and Schulze, 2012). The Warburg effect is the hallmark of cancer research play an important role in promoting the occurrence and development of tumors (Hanahan and Weinberg, 2011). It is the observation that most tumor cells still rely on aerobic glycolysis for energy even with adequate oxygen and nutrition. As such, it promotes rapid proliferation of cancer cells, cancer progression, and resistance to apoptotic cell death. PDAC cells adjust their metabolic pathways to meet the conditions needed for growth (Commisso et al., 2013; Chen et al., 2015; Mayerle et al., 2018).

Cognizant to this, studying glycolysis-related processes and key genes of pancreatic cancer may provide new insights that can aid in identification of potential targets for PDAC treatment. Herein, glycolysis genes that are significantly related to the prognosis of pancreatic cancer were identified. A genetic model based on these genes was then constructed and used to verify the unique prognostic markers of pancreatic cancer.

## Materials and Methods

### Data Acquisition and Processing

The gene expression profiles and clinical data of 148 patients with pancreatic ductal adenocarcinoma was obtained from the cancer and tumor gene profile database (TCGA)^1^. In the same line, pancreatic gene expression profile data of 168 normal individuals was obtained from Genotype-Tissue Expression database (GTEx)^2^. Gene expression profile data from both databases was then fused and corrected using “sva” package (Leek et al., 2019) of the R software (v3.61) as the training set.

### Gene Set Enrichment Analysis (GSEA) Analysis of Glycolysis-Related Pathways

Data of five pathways related to glycolysis was obtained from GSEA official website^3^. The five pathways were: GLYCOLYSIS_PATHWAY, HALLMARK_GLYCOLYSIS, GLY COLYSIS_GLUCONEOGENESIS, GLYCOLYTIC_PROCESS, and REACTOME_GLYCOLYSIS. Subsequent analysis of the training data was also done using GSEA.

### Differential Expression Analysis and Model Construction of Glycolysis Related Genes

Glycolysis pathways with a significantly close relationship with pancreatic cancer (*P* < 0.05) were selected based on the results of GSEA. The expression level of genes in these pathways was then summarized as glycolysis related genes (GRGs). The expression level of GRGs from the training set was subsequently extracted and the significant differences in the expression profiles (*P* < 0.05, logFC ≥ 1 or ≤ -1)between GRGs from the glycolysis pathways and those from the training set analyzed using the limma software package in the R v3.61 software. GRGs closely related to survival were further analyzed using the Cox risk regression analysis to screen out the most significant genes for construction of a GRGs model. The relative expression levels of the genes in the model were extracted and used to construct a heat map. In the same line, a receiver operating characteristic curve (ROC) was used to evaluate the accuracy of the model. The critical value of the model was then used as a basis to distinguish high-risk and low-risk groups.

### GRGs Model Verification

TCGA pancreatic ductal adenocarcinoma samples were randomly divided into two groups: a training and verification set. The GRGs model was then verified using single-factor and multi-factor COX proportional hazard analysis and survival analysis of the training set and verification set.

### Expression and Mutation of Model Genes

The expression levels of seven model genes in the training group were extracted using the limma package of the R software v3.61 and divided into the normal group and the tumor group based on the sample information. The expression data of both groups was then transformed to log2(TPM + 1) for differential expression analysis. The log2FC was defined as median (Tumor)—median (Normal). As such, the differential expression of genes was determined by comparing the values of log2FC. In addition, the immunohistochemical images of the seven genes in pancreatic cancer tissues and normal pancreatic tissues were searched in the Human Protein Atlas (HPA)^4^ to enable verification of differential gene expression between the two groups by immunohistochemical staining. Mutations in the seven model genes were identified using the mutation function in the cBioPortal website^5^.

### GRGs Model and Clinical Characteristics—Nomogram Diagram of the Model

The relationship between clinical characteristics and survival rate of patients in the training group was analyzed based on the model classification. A nomogram diagram was then constructed based on the GRGs model to evaluate the survival rates of patients with pancreatic cancer.

### Data Analysis

Data was expressed as mean ± standard deviation (x ± s), and compared using the student’s *t*-test. Subsequent survival analysis was performed using the Kaplan-Meier method. In addition, the ROC analysis was performed using the survivalROC program v1.0.3. The Cox proportional hazard regression model was used for both univariate and multivariate analysis. *P*-values less than 0.05 (*p* < 0.05) indicated that there were significant differences between groups. Values less than 0.01 (*p* < 0.01) indicated that the difference was highly significant.

## Results

### GSEA Analysis of Glycolysis-Related Pathways

**FIGURE 1:**
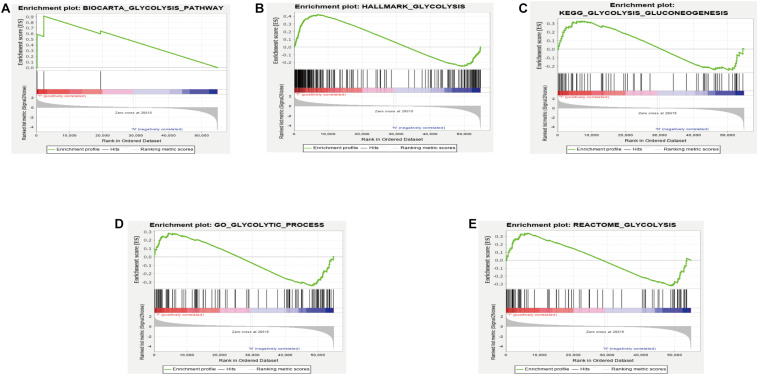
**(A)** GSEA analysis of the GLYCOLYSIS_PATHWAY. **(B)** GSEA analysis of HALLMARK_GLYCOLYSIS. **(C)** GSEA analysis of GLYCOLYSIS_GLUCONEOGENESIS. **(D)** GSEA analysis of GLYCOLYTIC_PROCESS. **(E)** GSEA analysis of REACTOME_GLYCOLYSIS.

GSEA analysis revealed that the genes in the training set were significantly enriched in the glycolysis pathway (Figure 1A) and the iconic glycolysis pathway (Figure 1B) (*p* < 0.05) (Figure 1 and Table 1).

**TABLE 1:** Details of GSEA results.

Name	Size	ES	NES	NOM *p*-val	FDR *q*-val
BIOCARTA_GLYCOLYSIS_PATHWAY	3	0.9109372	1.4394337	0.015086207	0.015086207
HALLMARK_GLYCOLYSIS	200	0.42210713	1.6729742	0.017857144	0.017857144
KEGG_GLYCOLYSIS_GLUCONEOGENESIS	62	0.3265195	1.2391948	0.19919518	0.19919518
GO_GLYCOLYTIC_PROCESS	106	–0.34641427	–1.3654215	0.14693877	0.14693877
REACTOME_GLYCOLYSIS	72	0.341253	1.2525212	0.22839506	0.22839506

### Differential Expression Analysis and Model Construction of Glycolysis Related Genes

**FIGURE 2:**
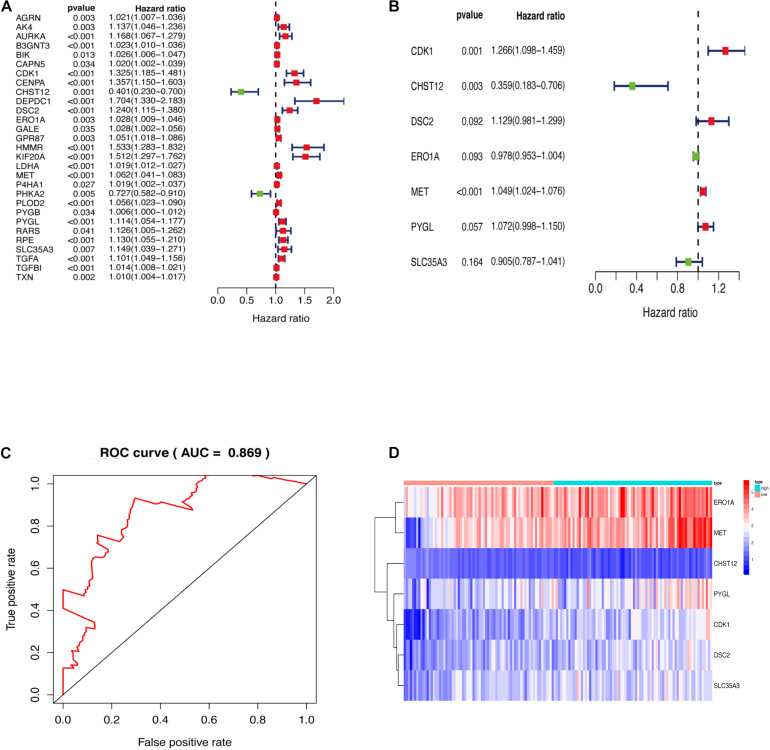
**(A)** GRGs associated with prognosis. **(B)** Key model GRGs. **(C)** Time-dependent ROC curve for GRGs in the training cohort. **(D)** Relative expression of model gene.

Genes enriched in the glycolysis and iconic glycolysis pathways were extracted from the training set for differential expression analysis. There were 108 differentially expressed GRGs. Among them, 29 GRGs were closely related to prognosis based on clinical survival time (Figure 2A). COX risk regression analysis further identified the seven optimal GRGs (CDK1, DSC2, MET, PYGL, CHST12, ERO1A, and SLC35A3) among the 29. A 10-fold cross-validation of the genes was then done and the prognostic model constructed. CDK1, DSC2, MET, and PYGL were found to be poor prognostic genes, while CHST12, ERO1A, and SLC35A3 were good prognostic genes (Figure 2B). The ROC curve of the model further revealed that the AUC (Area Under Curve) value was as high as 0.869 (Figure 2C). This was a strong indication that the model had high accuracy. The two groups are evaluated and the samples are divided into high and low risk groups after calculating the risk value according to the GRGs model. Figure 2D shows the expression of seven model genes in high and low risk groups.

### GRGs Model Verification

**FIGURE 3:**
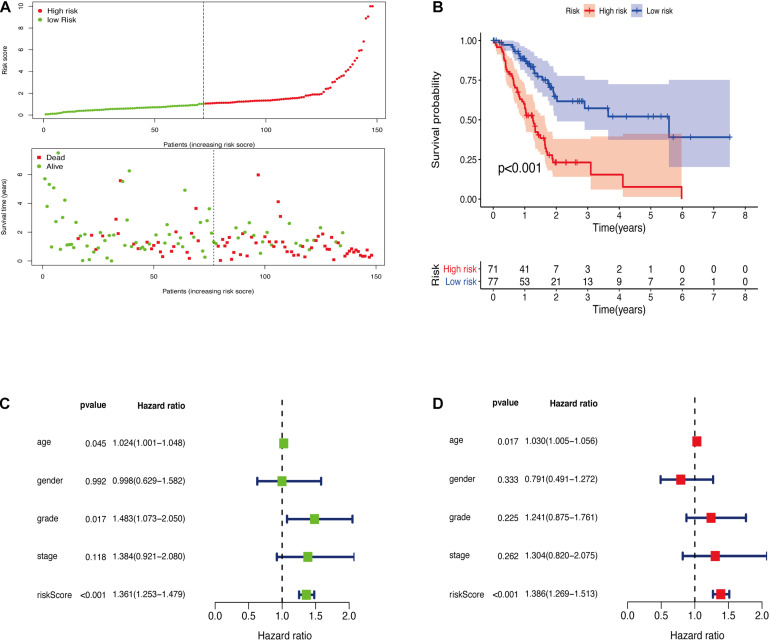
**(A)** The model divides the training set patients into low-risk or high-risk groups. **(B)** Kaplan Meier curve between high and low risk groups. **(C)** Training set single factor Cox regression analysis forest map. **(D)** Training set multivariate Cox regression analysis forest map.

**FIGURE 4:**
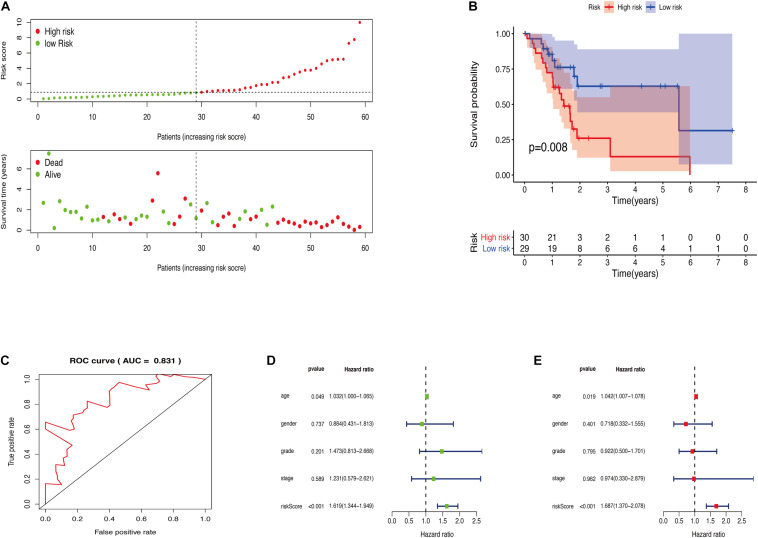
**(A)** The model divides the testing set patients into low-risk or high-risk groups. **(B)** Kaplan Meier curve between high and low risk groups. **(C)** Time-dependent ROC curve for GRGs in the testing cohort. **(D)** Testing set single factor Cox regression analysis forest map. **(E)** Testing set multivariate Cox regression analysis forest map.

The GRGs model was verified using data from the training group. The OS of the high risk group was significantly lower than that of the low risk group in the training set (Figures 3A,B; The green dot in the upper part of Figure 3A represents the patients with low risk, red represents the patients with high risk, the y-axis represents the risk score, and the dotted line represents the grouping. The green dot in the lower part of Figure 3A represents the alive patients in the group, the red represents the dead patients in the group, and the y-axis represents the survival time. We have marked the meaning of the green and red dots in the upper and lower part of Figure 3A in the upper left corner of the figure. Combining the upper and lower parts of Figure 3A, it can be clearly seen that the number of deaths in the high-risk group is significantly higher than that in the low-risk group.). In the same line, univariate risk regression analysis revealed that age, grade, and GRGs model were significantly correlated with prognosis (Figure 3C). Multivariate risk regression analysis further revealed that only age and GRGs model could be used as significant independent prognostic factors (Figure 3D). In addition, the survival analysis of the verification set demonstrated that the OS of patients in the high risk group was significantly lower than that of the low risk group (Figures 4A,B). The AUC value of the ROC curve of this model was more than 0.85 (Figure 4C) thus further confirming that the model had high accuracy. Univariate risk regression analysis of the validation set also confirmed that age and GRGs model were significantly correlated with prognosis (Figure 4D). Similarly, multivariate risk regression analysis of the validation set confirmed that only the GRGs model could be used as significant independent prognostic factors (Figure 4E).

### Expression and Mutation of Model Genes

**FIGURE 5:**
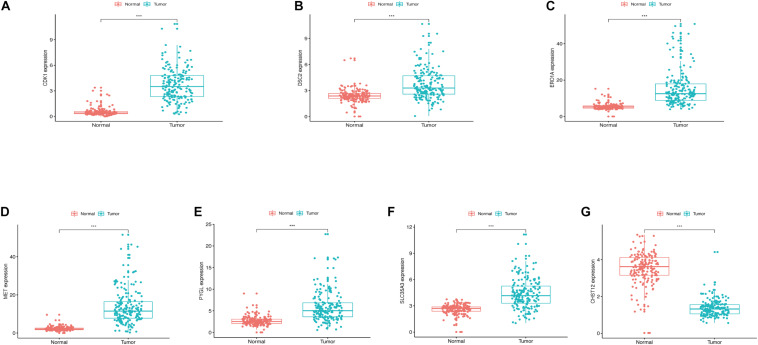
**(A)** Expression of CDK1 in PDAC and normal tissues. **(B)** Expression of DSC2 in PDAC and normal tissues. **(C)** Expression of ERO1A in PDAC and normal tissues. **(D)** Expression of MET in PDAC and normal tissues. **(E)** Expression of PYGL in PDAC and normal tissues. **(F)** Expression of SLC35A3 in PDAC and normal tissues. **(G)** Expression of CHST12 in PDAC and normal tissues. ***p < 0.001.

**FIGURE 6:**
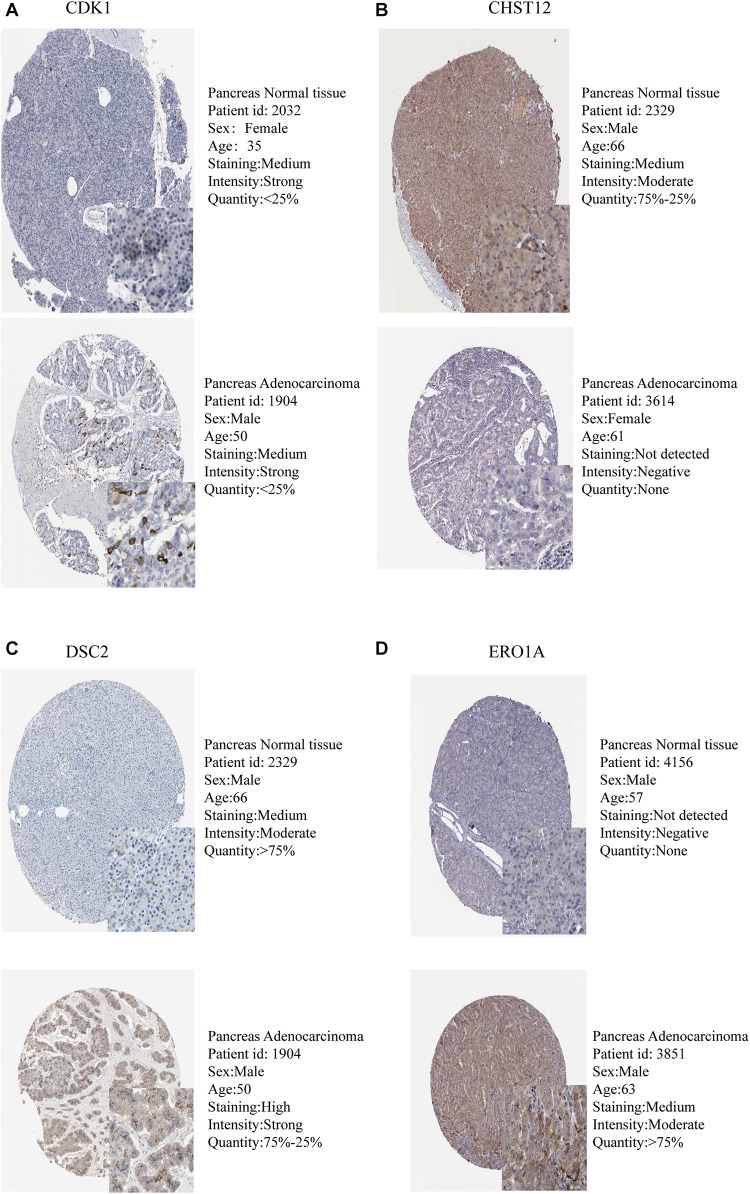
Validation of model GRGs IHC images obtained from the Human Protein Atlas database **(A–D)**.

**FIGURE 7:**
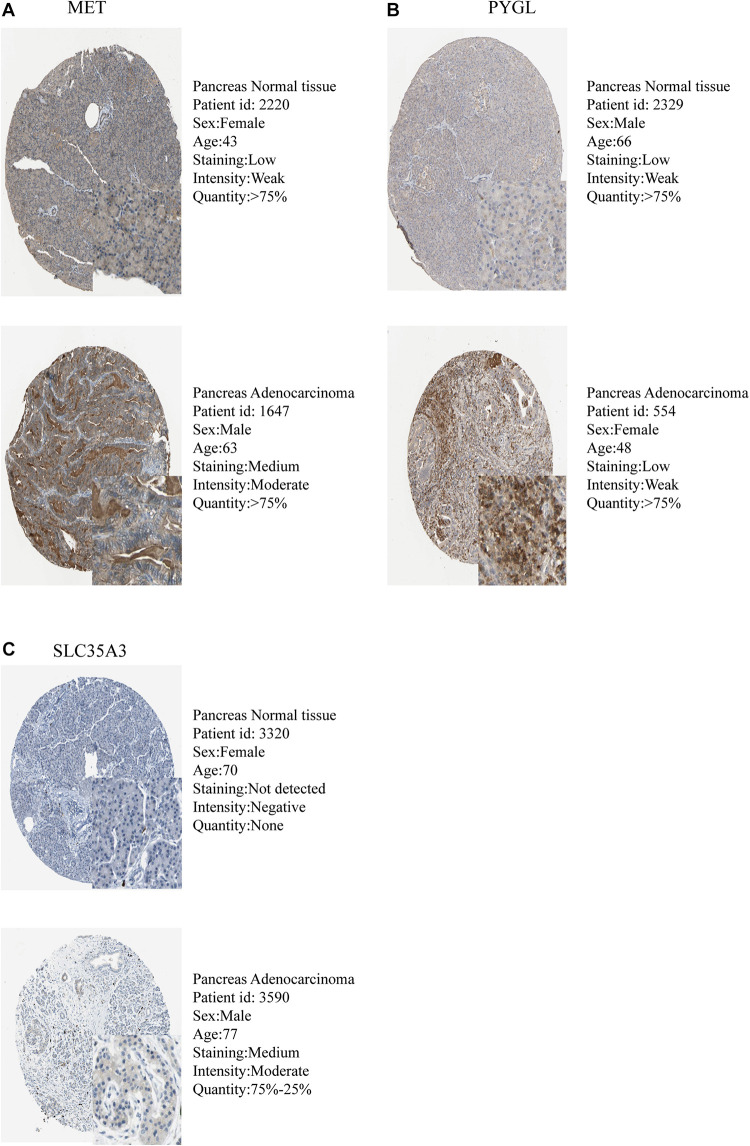
Validation of model GRGs IHC images obtained from the Human Protein Atlas database **(A–C)**.

**FIGURE 8:**
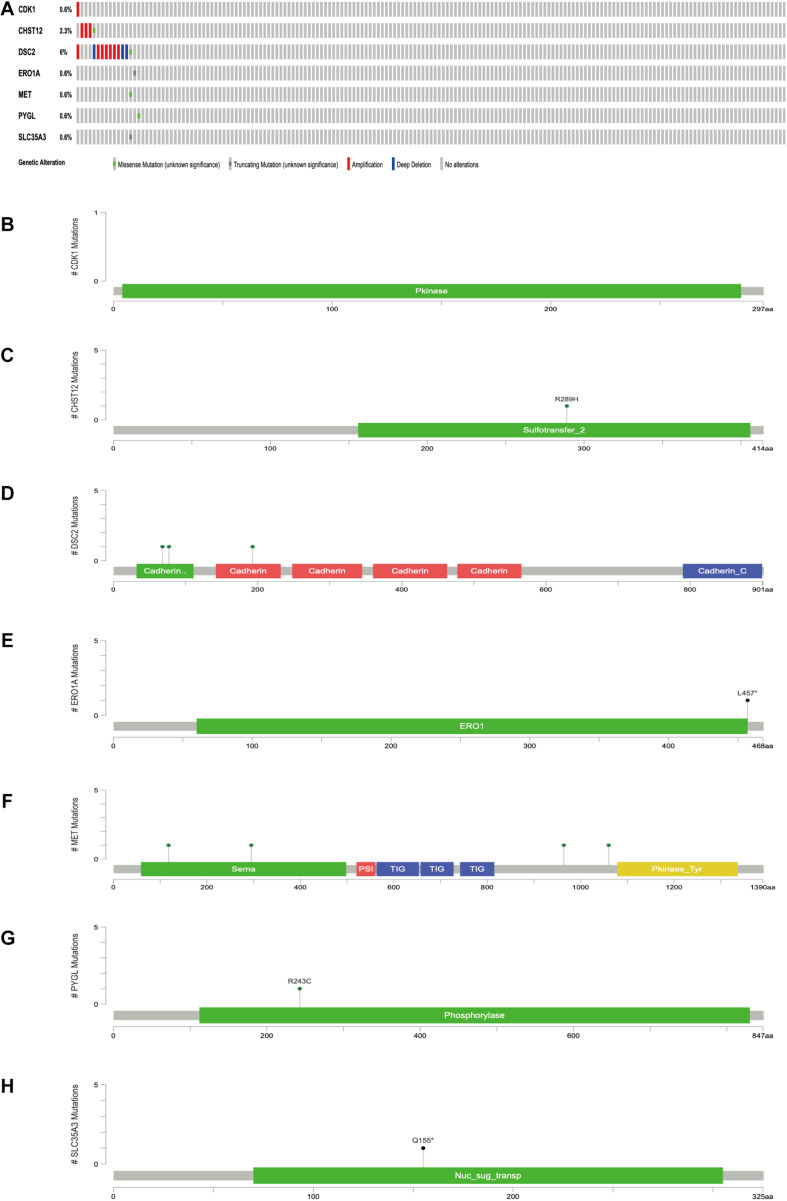
**(A)** Mutations of model genes. **(B)** Mutation of CDK1. **(C)** Mutation of CHST12. **(D)** Mutation of DSC2. **(E)** Mutation of ERO1A. **(F)** Mutation of MET. **(G)** Mutation of PYGL. **(H)** Mutation of SLC35A3. *p < 0.05.

The expression levels of the seven model genes in the training set were analyzed. CDK1 (Figure 5A), DSC2 (Figure 5B), ERO1A (Figure 5C), MET (Figure 5D), PYGL (Figure 5E), and SLC35A3 (Figure 5F) were highly expressed while CHST12 (Figure 5G) was decreased in pancreatic cancer. These findings were verified by analyzing the immunohistochemical images of the seven genes in pancreatic cancer and normal pancreatic tissues obtained from the HPA website^6^ (Uhlén et al., 2015). Figures 6, 7 shows the immunohistochemical images of the seven genes obtained from the Human Protein Atlas version 19.3. Identification of mutations in model genes further revealed that DSC2 and CHST12 had higher mutation rates compared to CDK1, ERO1A, MET, PYGL, and SLC35A3 (Figures 8A–H).

### GRGs Model and Clinical Characteristics—Nomogram Diagram of the Model

**FIGURE 9:**
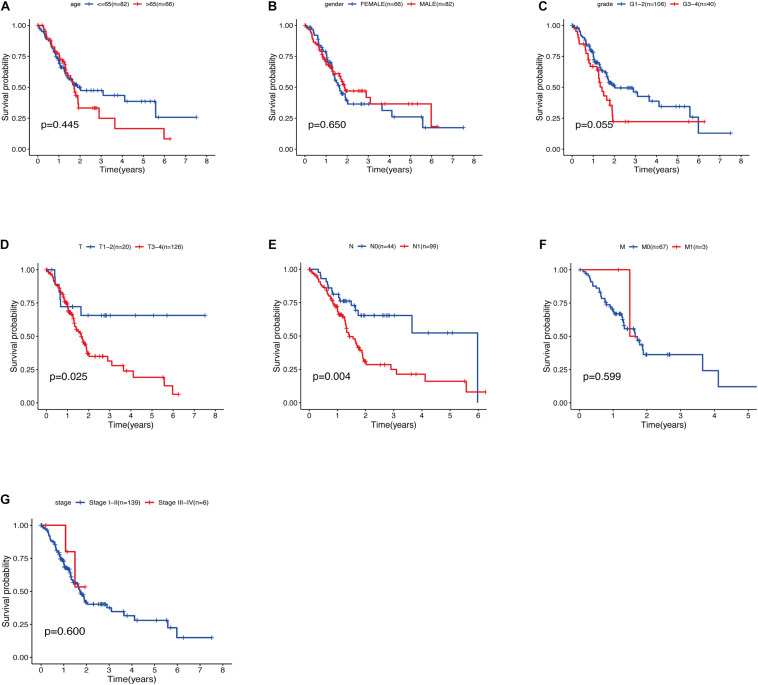
**(A)** Relationship between age and survival of patients with PDAC. **(B)** Relationship between gender and survival of patients with PDAC. **(C)** Relationship between grade and survival of patients with PDAC. **(D)** Relationship between T stage and survival of patients with PDAC. **(E)** Relationship between N stage and survival of patients with PDAC. **(F)** Relationship between M stage and survival of patients with PDAC. **(G)** Relationship between stage and survival of patients with PDAC.

**FIGURE 10:**
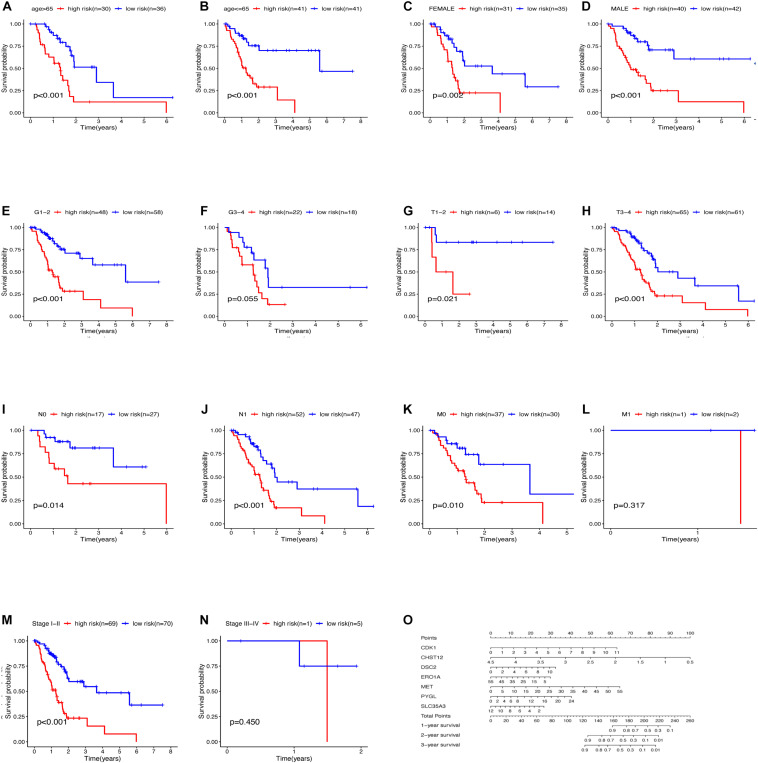
Relationship between clinical features and survival of patients with pancreatic cancer after model grouping **(A–N)**. **(O)** Nomogram of the GRGs model.

The relationship between clinical traits (Table 2) and survival rates of pancreatic cancer patients was analyzed. The analysis revealed that only T stage (Figure 9D) and N stage (Figure 9E) were significantly related to the survival rate of patients. However, age, gender, grade, stage, and survival rate were not statistically significant (Figures 9A–C,F,G). The clinical traits were divided into groups based on the GRGs model and the subsequent survival rates of patients in each group analyzed. The GRGs model could explicitly distinguish 11 patients with different clinical characteristics (Figures 10A–C,G,H,J,K,M). Nonetheless, distinguishing patients using G3-G4 (Figure 10F), M1 (Figure 10L), and Stage III-IV (Figure 10N) was not significant. The GRGs model was further used to construct a nomogram to intuitively evaluate the prognosis of patients with pancreatic cancer (Figure 10O).

**TABLE 2:** TCGA clinical information.

Variable	Count
**Age**	
≤65	96
>65	89
**Gender**	
Female	83
Male	102
**Grade**	
G1	32
G2	97
G3	51
G4	2
GX	3
**Stage**	
I	21
II	152
III	4
IV	5
Missing	3
**Stage**	
T1	7
T2	24
T3	148
T4	4
TX	1
Missing	1
**Stage N**	
N0	50
N1	130
NX	4
Missing	1
**Stage M**	
M0	85
M1	5
MX	95

## Discussion

Pancreatic ductal carcinoma (PDAC) is one of the most rapidly progressive and lethal malignant tumors (Siegel et al., 2020). Its 5-year survival rate is less than 6%. Moreover, the overall survival rate of PDAC patients has not changed significantly despite numerous tumor molecular studies being conducted in the last 20 years (Rahib et al., 2014; Makohon-Moore and Iacobuzio-Donahue, 2016). There are no significant symptoms at the initial stage of the disease because of the special anatomical location and physiological function of the pancreas. Moreover, routine examination is difficult to clearly show the state of the pancreas. This causes most PDAC patients to be diagnosed at an advanced stage. It is therefore important to find effective biological targets for prediction and treatment of PDAC.

Notably, PDAC tissues often develop connective tissue hyperplasia thereby forming a significantly dense matrix on the pancreas because of its location (Chu et al., 2007). The dense matrix can compress the space between tumor and normal pancreas consequently leading to nutritional deficiency and high hypoxia (Ma et al., 2011; Hingorani, 2014). In addition, the microenvironment of mesenchymal tumors can greatly promote progress of PDAC. The Warburg effect also plays a key role in PDAC cell proliferation and survival (Jiang et al., 2017; Zhang et al., 2019). Cognizant to this, studying PDAC glycolysis-related pathways and genes is a potential method to find possible biological targets for prediction, diagnosis and treatment of PDAC. To date, only a few studies have been done to decipher the relationship between macrophages, organic ferritin, miRNAs and glycolysis, and their role in pancreatic cancer (Rademaker et al., 2018; Ye et al., 2018; Yang et al., 2019). Moreover, there are no studies on the PDAC glycolysis pathway as well as the use of clinical patient sample sequencing to analyze GRGs related to the survival rate of PDAC patients.

Herein, PDAC data from TCGA and GTEx databases was integrated and analyzed to identify the correlation between the data and glycolysis. Seven GRGs: CDK1, DSC2, MET, PYGL, CHST12, ERO1A, and SLC35A3 were selected to construct the prognostic model related to the overall survival rate of patients with pancreatic cancer. The AUC value of the model was greater than 0.85 in both the TCGA training group and the verification group. Moreover, the OS of the high-risk group was significantly lower than that of the low-risk group in both the training group and verification group based on the seven GRGs. This was an indication that the seven GRGs had a good discrimination effect on the samples. Expression analysis further revealed that CDK1, DSC2, ERO1A, MET, PYGL, and SLC35A3 were highly expressed in pancreatic cancer while CHST12 was highly expressed in normal pancreatic tissues. These findings suggested that the six GRGs highly expressed in pancreatic cancer were related to poor progression of PDAC while CHST12 was related to good prognosis. Previous studies postulate that high expression of CDK1 is related to poor prognosis of PDAC (Piao et al., 2019). In addition, interactions between CDK1 and KRAS have a synergistic strong lethal effect (Costa-Cabral et al., 2018). Expression of DSC2 is significantly correlated with the survival time of high-grade colorectal cancer patients (Knösel et al., 2012). In addition, Saunus et al. (2017). Studied brain metastasis samples including lung cancer, breast cancer, esophageal cancer and melanoma, and found that the mutation rate of DSC2 is very high, and it may be a new marker in the development of brain metastasis. Our results also show that the mutation rate of DSC2 is high in PDAC, which may be one of the reasons why DSC2 plays a role in PDAC. MET is one of the important carcinogenic receptors (Omote et al., 2020). As such, high expression of MET indicates rapid progression of lymphoma in gastric mucosa-associated lymphoid tissues (Xu et al., 2020). In the same line, PYGL is a key gene in the glycolysis pathway. Expression of PYGL increases the reactive oxygen species thereby inducing p53-dependent senescence and significant damage to tumorigenesis *in vivo* (Favaro et al., 2012). PYGL was found to be highly associated with disease recurrence in a study of childhood acute lymphoblastic leukemia. Moreover, the risk of recurrence of the C allele in the PYGL gene was 3.6 times higher than that of the T allele (Yang et al., 2012). These findings were similar to those of this study (Figure 8G). Yan et al. (2019) and Yan et al. (2019) reported that high expression of ERO1A in cholangiocarcinoma (CCA) was associated with clinical and pathological stages of CCA. It is also postulated that ERO1A promotes growth, migration and invasion of tumor cells through the Wnt/catenin pathway (Han et al., 2018). SLC35A3 is a pathogenic gene of T-cell lympho-blastoma (López-Nieva et al., 2019). In a toxicity test of amphetamine, most transcripts specific to T cells were found to decrease by 50–70% after exposure to amphetamine. CHST12 was the only exception which strongly suggested that it played a protective role in the event of damage. Similarly, CHST12 was found to be a favorable prognostic factor for pancreatic cancer in this study. Generally, the highly expressed model genes in PDAC play a key role in PDAC and other tumors. As such, they are closely related to tumor progression and patient survival. Nonetheless, decreased expression of the CHST12 gene in tumor cells produces an anti-tumor effect by acting on the immune cells.

TNM staging and clinical staging are currently the most widely used methods to evaluate tumor malignant potential and disease progression, but this method also has some shortcomings, for example: after radical resection of patients with the same disease and the same stage, the patient’s prognosis The situation will also be very different. In addition, this staging method largely depends on the location, size, lymph node invasion and whether there is distant metastasis of the tumor, and fails to take into account the heterogeneity of various tumors, age, gender and other clinical information. Our results also showed that only the T and N stages of the analyzed PDAC samples were significantly associated with patient survival (Figures 10D,E). The patient’s survival rate is not significantly related to the age, sex, grade and stage of the cancer. This result may be due to the high degree of malignancy of pancreatic cancer and the short survival time of patients, resulting in the lack of statistically significant differences in the analysis of these clinical features and survival, but it is enough to see that these evaluation methods have significant deficiencies Place. However, we use the GRGs model constructed in this study to predict the prognosis of PDAC samples, 11 clinical groups showed a close correlation with patients’ survival after grouping using the GRGs model. These findings demonstrated that the GRGs model had high accuracy and could better predict the prognosis of PDAC patients than the current generally applicable grading and staging methods.

Moreover, most of the previous studies focused on the role of a certain gene in PDAC, and most of them studied the effect of genes on the function of PDAC cells, for example: Yang et al. (2020) found that chlorogenic acid can inhibit cell bioenergy by regulating the c-Myc-TFR1 axis, thereby inhibiting pancreatic cancer. The study by Wang et al. (2020) shows that p38γ links KRAS oncogene signal transduction and Warburg effect through PFBBF3 and Glut2 to promote the occurrence of pancreatic cancer. But our study creatively studied the relationship between glycolysis-related genes (GRGs) and the clinical risk of PDAC patients, and the constructed model can predict the prognosis of PDAC patients well. Evidently, the GRGs model constructed herein can play an important role determining the occurrence and development of PDAC as well as the prognosis of PDAC patients.

There are also some deficiencies in our research. Although we select data samples from two databases for analysis, and evaluated the results by immunohistochemistry, this is still a retrospective analysis. If we can carry out a prospective study by using GRGs model to conduct prospective research in multi center, it will be more convincing.

## Conclusion

A glycolysis gene model which is closely related to the prognosis of pancreatic ductal adenocarcinoma patients was successfully constructed. The model contains seven GRGs whose biological functions are closely related to the occurrence and development of pancreatic ductal adenocarcinoma. Cognizant to this, these GRGs may be potential targets for predicting or diagnosing PDAC. They can also be regulated to improve the prognosis of PDAC patients.

## Data Availability Statement

Publicly available datasets were analyzed in this study. This data can be found here: The Cancer Genome Atlas link: https://portal.gdc.cancer.gov; Human Protein Atlas available from http://www.proteinatlas.org; cBioPortal repository available from http://www.cbioportal.org/.

## Author Contributions

HN: research design and drafting the manuscript. CL: literature search. KL: helping to draft the manuscript. JX: helping to design the research. XW: review and revision of the manuscript and writing guidance. X-XC: helped to revised the manuscript. All authors contributed to the article and approved the submitted version.

## Conflict of Interest

The authors declare that the research was conducted in the absence of any commercial or financial relationships that could be construed as a potential conflict of interest.

## Footnotes

1 http://www.tcga.org

2 https://www.gtexportal.org

3 http://software.broadinstitute.org/gsea

4 https://www.proteinatlas.org/

5 http://www.cbioportal.org/

6 http://www.proteinatlas.org
