# Corrigendum: Seven Glycolysis-Related Genes Predict the Prognosis of Patients With Pancreatic Cancer

**DOI:** 10.3389/fcell.2021.695280

**Published:** 2021-06-28

**Authors:** Han Nie, Cancan Luo, Kaili Liao, Jiasheng Xu, Xue-Xin Cheng, Xiaozhong Wang

**Affiliations:** ^1^Department of Vascular Surgery, The Second Affiliated Hospital of Nanchang University, Nanchang, China; ^2^Department of Hematology, The Second Affiliated Hospital of Nanchang University, Nanchang, China; ^3^Jiangxi Province Key Laboratory of Laboratory Medicine, Department of Clinical Laboratory, The Second Affiliated Hospital of Nanchang University, Nanchang, China; ^4^Jiangxi Province Key Laboratory of Laboratory Medicine, The Second Affiliated Hospital of Nanchang University, Nanchang, China

**Keywords:** glycolysis-related genes, pancreatic ductal carcinoma, prognosis, model, predict

In the original article, we neglected to include the funder National Natural Science Foundation of China, 81860034 to Xiaozhong Wang. The corrected Funding Statement appears below:

“We would like to acknowledge the funder National Natural Science Foundation of China (No. 81860034 to XW).”

In the published article, there was an error in affiliation **3**. Instead of “Department of Clinical Laboratory, The Second Affiliated Hospital of Nanchang University, Nanchang, China,” it should be “Jiangxi Province Key Laboratory of Laboratory Medicine, Department of Clinical Laboratory, the Second Affiliated Hospital of Nanchang University, Nanchang, China.”

Author Xue-Xin Cheng was not included as an author in the published article. The author's affiliation is “Jiangxi Province Key Laboratory of Laboratory Medicine, the Second Affiliated Hospital of Nanchang University, Nanchang, China.” Xue-Xin Cheng is a co-corresponding author of the article. The correspondence section has been updated and the corrected Author Contributions Statement appears below.

“HN: research design and drafting the manuscript. CL: literature search. KL: helping to draft the manuscript. JX: helping to design the research. XW: review and revision of the manuscript and writing guidance. X-XC: helped to revised the manuscript. All authors contributed to the article and approved the submitted version.”

The authors apologize for these errors and state that this does not change the scientific conclusions of the article in any way. The original article has been updated.

